# Prevalence of dental caries and associated factors of detention center inmates in South Korea compared with Korea National Health and Nutrition Examination Survey (KNHANES) respondents: a retrospective study

**DOI:** 10.1186/s12903-022-02405-w

**Published:** 2022-09-05

**Authors:** Ilkwang Hwang, Kyungtaek Park, Hee-Kyung Park

**Affiliations:** 1 Department of Oral Medicine and Diagnosis, School of Dentistry and Dental Research Institute, Seoul National University, #101, Daehak-ro, Jongro-Gu, Seoul, 03080 Korea; 2 Institute of Health and Environment, Seoul National University, Seoul, Korea

**Keywords:** Correctional institution, Dental caries, Diagnostic self-evaluation, DMF index, National Health and Nutrition Examination Survey, Oral health, Statistical factor analysis

## Abstract

**Background:**

Correctional institution inmates have reduced access to dental care; however, a quantitative assessment of their oral health condition has not yet been performed in South Korea. Therefore, this study aimed to assess dental caries and compare the prevalence of dental caries and associated factors between inmates and the general South Korean population.

**Methods:**

The dental records of two detention centers in South Korea were retrospectively analyzed to assess the clinical oral health condition of inmates using the Decayed, Missing, and Filled Teeth (DMFT) index and self-reported questionnaire. These data were compared with similar data obtained from the Korea National Health and Nutrition Examination Survey (KNHANES) for the general South Korean population.

**Results:**

In total, 642 inmates were analyzed and compared with 13,345 KNHANES participants in the KNHANES. The inmate and KNHANES groups demonstrated significant intergroup differences, with a higher prevalence of untreated caries, DMFT, decayed teeth (DT), and missing teeth (MT) values among the inmates. The prevalence of untreated caries decreased according to the history of dental pain in the inmate group but increased in the KNHANES group. The decrease in DMFT with a history of dental pain was significant only in the inmate group. Furthermore, self-rated oral health was significantly associated with prevalence of untreated caries, DMFT, DT, MT, and filled teeth (FT) in the inmate group but with prevalence of untreated caries, DMFT, DT, and MT in the KNHANES group. It was found that this is because there is an interaction effect by the group.

**Conclusions:**

The oral health of the inmate group was significantly poorer than that of the general group. Since DMFT, DT, MT, and FT values and prevalence of untreated caries in the inmate group were significantly related to their self-rated oral health, suggesting that self-rated oral health should be incorporated into the dental health screenings of correctional institution inmates.

**Supplementary Information:**

The online version contains supplementary material available at 10.1186/s12903-022-02405-w.

## Background

In South Korea, various social systems operate to promote public oral health, and the accessibility to individual dental care is increasing. Despite this improved accessibility, vulnerable groups in South Korea sometimes require specialized dental care [1]. Studies have shown that vulnerable groups do not always have ready access to dental services due to limited resource availability [2]. There are 54 local correctional institutions in South Korea, including 40 prisons (including one private prison), 11 detention centers, and three branches. Each local correctional institution has an affiliated clinic that provides health care to inmates. Dental care is generally performed at these affiliated clinics, and all dentists working at these clinics are either public health dentists or external dentists without any permanent (regular) dental officers. Dental treatment is provided free of charge and within the capacity of in-house facilities and resources.

According to Article 38 of the Administration and Treatment of Correctional Institution Inmates Act (ATIA), some local correctional institutions also offer care from dentists who are invited to provide medical treatment at the inmate’s own expense. However, there are no guidelines for treatment at one’s own expense, and finding external doctors who will provide this type of treatment is challenging. According to Article 37 of the ATIA, external medical institutions can also be used for inmate care in emergencies or for more complex cases. However, visits to external medical institutions are difficult to arrange due to insufficient personnel and the need for consultations with security departments. According to nationwide data from Korea Correctional Service [3], only 1096 inmates used external medical institutions for dental treatment in 2018.

Dental caries are associated with socioeconomic disparities because they disproportionately affect low-income and racially or ethnically diverse populations [4]. Inmates at correctional institutions are more likely to have poorer oral health than non-incarcerated individuals and often require more intensive care and treatment [5–10]. Dental indifference and self-rated oral health affect the oral health-related quality of life of prisoners [11].

Several studies have been conducted on various associated factors affecting dental caries [12–18]. In terms of the effects of self-rated oral health on dental caries, a few studies have reported that the number of missing and decayed teeth is proportional to the probability of oral health being self-evaluated as poor. Additionally, the presence of more unreplaced teeth negatively affects the results of oral health evaluation [12]. Conversely, the greater the number of filled teeth, the more likely it is to perceive oral health as good [13, 14]. Adults with multiple dental caries suffer from chewing deficiencies [15]. According to a meta-analysis, the habit of tooth-cleaning was not significantly associated with dental caries, and there is high non-uniformity in the evaluation of the tooth cleaning habit [16]. Since biofilm formation is related to periodontitis and dental caries, the effect of periodontal disease, diabetes, cardiovascular disease, and gingival bleeding on dental caries should be simultaneously considered [17]. The progression of dental caries and periodontal diseases involves multiple microbial interactions caused by various stressors [18].

Several studies have been conducted on the dental caries of prisoners in other countries [5–10]; however, there are few studies on the factors affecting these dental lesions. In addition, many studies did not consider confounders, such as general health, oral hygiene habits, and perceived needs. Moreover, there have been no quantitative studies assessing the general oral health of inmates in South Korea. Therefore, this study aimed to assess dental caries and compare the prevalence of dental caries and associated factors between inmates and the general population in South Korea.

## Methods

### Study population

The study was approved by the Institutional Review Board of the School of Dentistry at Seoul National University, Seoul, Republic of Korea (IRB No. S-D20200018). All methods were performed in accordance with the relevant guidelines and regulations by including a statement in the declaration section under the ethical approval and consent for participation section.

Dental appointments were performed from October 7, 2019 to February 29, 2020, at two detention centers in South Korea, including the Seoul Detention Center and the Seoul Eastern Detention Center (Additional file 1: Appendix 1). Dental records were compiled by the authors in March 2020. A retrospective analysis of the dental records of 642 inmates was performed. Informed consent was waived by the intstitutional review board because of the retrospective study design. Separate serial numbers were coded and assigned to the inmates without collecting identifiable information. Only the researchers had access to the collected data, and the data were kept in encrypted files protected by the research manager for three years after the study’s completion.

For comparison, raw data related to oral examinations were similarly obtained from the Korea National Health and Nutrition Examination Survey (KNHANES) VII (2016–2018) conducted by the Korea Disease Control and Prevention Agency (KDCA) [19]. The target population of the survey was all non-institutionalized civilian South Korean individuals one year of age or older. The survey employed stratified multistage probability sampling units based on the geographical area, sex, and age, which were determined based on the household registries of the National Census Registry—the most recent 5 years of the national census in South Korea [20]. Using the census data, 192 primary sampling units (PSU) were selected annually across South Korea [21]. Raw data for 16,489 people surveyed between 2016 and 2018 were released most recently in the KNHANES VII.

According to the South Korean Criminal Act, individuals below age 14 cannot be admitted into correctional institutions. In addition, the growth of permanent teeth (except for wisdom teeth) is usually complete after age 14. Therefore, KNHANES data from 2523 people below age 14 were excluded. In addition, the survey excluded data from 621 people aged 80 or older, as top-coding was used to de-identify the elderly population. Therefore, 13,345 individuals were included in the KNHANES group.

### Data collection

For detention center inmates, clinical examination was performed using a dental chart during dental appointments conducted by a public health dentist (I. Hwang; Additional file 1: Appendix 1). Examinations were performed in a dental clinic in the correctional institutions equipped with basic dental equipment such as; a dental chair, artificial light, and intra-oral mirror. Additionally, a brief self-reported questionnaire was applied together with the clinical examination to quickly and comprehensively assess a patient’s oral health condition during the appointment. The questionnaire was not previously validated and was created by the authors. The questionnaire items were the same as those used in the oral examination conducted by the National Health Insurance Service which is used nationwide in health checkups based on Article 52 of the National Health Insurance Act. However, questions on diet, smoking, and the use of fluoride toothpaste were excluded because all participants were expected to provide the same response, as these aspects were controlled at the correctional institution. Unfortunately, there are no such clinical examination data matching this questionnaire for the general population.

At the time of writing, there are no existing protocols for dental triage in this setting; therefore, inmates in this study were treated sequentially based on their order of application. However, an inmate’s appointment was prioritized if an emergency was reported through an employee or a referral was made by a medical doctor.

The determination of epidemiological indices of dental caries such as the Decayed, Missing, and Filled Teeth (DMFT) index, the decayed teeth (DT) index, the missing teeth (MT) index, and the filled teeth (FT) index was based on the criteria of the KNHANES protocol, which are modeled after on the World Health Organization criteria [22]. While counting the DT, cavitated lesions and filled crown with caries were included. According to these guidelines, the assessments were made only through history taking and visual inspection, without the use of radiographs. The prevalence of untreated caries was determined by calculating the proportion of participants with any DT. An example of the dental records form used during the dental appointments is provided in the Additional file 1: Appendix 1.

Since 2007, the oral health of the general population of South Korea has been assessed using KNHANES (KNHANES has been implemented since 1998). Specifically, the KDCA conducts various oral examinations, including three self-reported oral health questions, oral/prosthodontic status tests (e.g., dental caries), treatment requirement tests, periodontal tests, and dental fluorosis tests. Since the DMFS index was used during the KNHANES collection phase, this information was converted to the DMFT index for comparison with inmate data.

### Statistical analysis

A contingency table was constructed to summarize the age and sex distribution by population group, and a chi-square test was performed to determine the difference in age and sex distribution between the two groups.

To examine the association between independent variables and the prevalence of untreated caries, a binary logistic regression analysis on a generalized linear model was used.

Assuming that the epidemiological indices of dental caries such as DMFT, DT, MT, and FT have a natural number of data, we can apply Poisson's regression analysis or negative binomial regression analysis on a generalized linear model. Based on the use of the Akaike information criterion, negative binomial regression analysis was considered more suitable. This regression analysis was similarly used in a previous study on dental caries [23].

The self-reported questionnaire in the inmate group was analyzed. To provide a comprehensive evaluation of oral health, the self-reported questionnaire included questions related to general health, perceived oral health, and oral health habits. For general health, a history of diabetes and cardiovascular diseases was considered. For perceived oral health, the following 11 questions were asked: “How long has it been since you last visited the dentist?” “Have you been uncomfortable chewing in the last three months?” “Have you felt pain in the last three months? (history of dental pain),” “Have your gums bled during the last three months? (bleeding of gums),” “What is your perception regarding your mouth condition when you evaluate yourself? (self-rated oral health).” Regarding oral health habits, the following questions were asked: “Have you ever learned how to brush your teeth?” “How many times do you brush your teeth in a day?” “How many times have you brushed your teeth right before going to bed in the last week?” and “How often do you use dental floss?” All variables from the questionnaire were included in the multivariable models based on their association with dental caries, as reported in the literature. Variable selection was not applied from the analysis even if it was insignificant [24]. A generalized linear model was used on all variables including sex, age, and the 11 questions as covariates, and a binary logistic regression analysis for the prevalence of untreated caries and negative binomial regression analysis for DMFT, DT, MT, and FT were performed.

Similarly, the individuals in the KNHANES group also received self-reported questionnaire. This questionnaire comprised two questions: “Have you experienced a toothache in the past year?” and “What is your perception of your mouth condition when evaluating yourself?” Moreover, a generalized linear model was used on all variables including sex, age, and the two questions as covariates, and a binary logistic regression analysis for the prevalence of untreated caries and negative binomial regression analysis for DMFT, DT, MT, and FT were performed.

A comparison of the prevalence of untreated caries and epidemiological indices of dental caries between the two groups was conducted. Among the data of the two groups, a history of dental pain during the past three months was assessed in the inmate group, and during the past one year in the general population group. The duration of interest differed between the two groups; however, we considered the responses comparable since they both addressed whether the patients or respondents had experienced general pain before visiting the clinic. Ultimately, we found four common variables: sex, age, history of dental pain, and self-rated oral health. Variable selection was not applied as the same above, and analysis was performed including all common variables. Thus, to compare the prevalence of untreated caries and epidemiological indices of dental caries between the two groups, a generalized linear model was used by adjusting for sex, age, history of dental pain, and self-rated oral health as covariates, which employed a binary logistic regression analysis for the prevalence of untreated caries and negative binomial regression analysis for DMFT, DT, MT, and FT.

In addition, to separately investigate the interaction effect of sex, age, history of dental pain, or self-rated oral health on the prevalence of untreated caries and epidemiological indices of dental caries by the group, we checked for the interaction effects between “group by sex,” “group by age,” “group by history of dental pain,” and “group by self-rated oral health,” with adjustments for the effects of sex, age, group, history of dental pain, and self-rated oral health. A generalized linear model was used, which employed a binary logistic regression analysis for the prevalence of untreated caries and negative binomial regression analysis for DMFT, DT, MT, and FT were performed.

Statistical analyses were performed using R software version 4.0.2 (R Foundation for Statistical Computing, Vienna, Austria). Statistical significance was set at a *p* value of < 0.05.

## Results

### Participants

In total, 642 inmates were analyzed, with 344 from the Seoul Detention Center and 298 from the Seoul Eastern Detention Center. Among these, 592 (92%) were men, and 50 (8%) were women. Of the 13,345 KNHANES participants, 5941 (45%) were men, and 7404 (55%) were women. The composition of each age group is shown in Table 1. There was a significant difference in sex and age between the two groups. (sex, chi-square value = 557.83, *p* < 0.001; age, chi-square value = 190.04, *p* < 0.001).

**Table 1:** Descriptive statistics for the study population

Age group (years)	Inmates (n = 642)			KNHANES (n = 13,345)		
	Men	Women	Total	Men	Women	Total
14‒19	11	1	12 (2%)	463	435	898 (7%)
20‒29	136	9	145 (23%)	685	746	1431 (11%)
30‒39	133	6	139 (22%)	938	1164	2102 (16%)
40‒49	125	11	136 (21%)	1041	1378	2419 (18%)
50‒59	120	10	130 (20%)	1064	1429	2493 (19%)
60‒69	55	11	66 (10%)	1013	1243	2256 (17%)
70‒79	12	2	14 (2%)	737	1009	1746 (13%)
Total	592 (92%)	50 (8%)	642 (100%)	5941 (45%)	7404 (55%)	13,345 (100%)

There was a significant difference in sex and age between the two groups. (Sex, Chi-square value = 557.83, *p* < 0.001; age, Chi-square value = 190.04, *p* < 0.001)

*KNHANES* Korea National Health and Nutrition Examination Survey

### Self-reported questionnaire responses in the inmate group and the KNHANES group

The questionnaire included 11 questions. When asked how long it had been since he/she had last visited the dentist, the detention center inmates reported an average of 27.1 months, with a median of 12.0 months. In total, 12.1% reported diabetes and 8.1% reported cardiovascular diseases. Further, 75.2% had a history of dental pain, 74.8% experienced bleeding gums, and 79.8% had discomfort while chewing. Only 4.7% of inmates reported that their oral health was “good”, and none reported it as “very good.” Inmates reported brushing their teeth 3.0 times per day (standard deviation = 0.9) on average. Other responses are shown in Table 2.

**Table 2:** The distribution of responses to self-reported questionnaire items in the study population

Variable	Inmate Patients (n = 642) n(%)		KNHANES (n = 13,345) n(%)	
	Total Answer	Missing data	Total Answer	Missing data
*Time since last dental visit (month)*	Mean/SD			
	27.1 (52.9)	2 (0.0%)		
*Diabetes*				
Yes	75 (12.1%)	22 (3.4%)		
No	545 (87.9%)			
*Cardiovascular disease*				
Yes	50 (8.1%)	24 (3.7%)		
No	568 (91.9%)			
*Discomfort while chewing*				
Yes	495 (79.8%)	22 (3.4%)		
No	90 (14.5%)			
Do not know	35 (5.6%)			
*Previous training on tooth brushing*				
Yes	332 (54.0%)	27 (4.2%)		
No	283 (46.0%)			
*Number of times teeth are brushed in a day*	Mean/SD			
	3.0 (0.9)	2 (0.0%)		
*Brushing of teeth before sleep*				
Always	298 (48.1%)	22 (3.4%)		
Usually	174 (28.1%)			
Sometimes	111 (17.9%)			
Rarely	37 (6.0%)			
*Use of dental floss*				
Always	79 (12.8%)	23 (3.6%)		
Usually	87 (14.1%)			
Sometimes	171 (27.6%)			
Rarely	282 (45.6%)			
*Bleeding gums*				
Yes	464 (74.8%)	22 (3.4%)		
No	137 (22.1%)			
Do not know	19 (3.1%)			
*History of dental pain*				
Yes	466 (75.2%)	22 (3.4%)	4165 (31.2%)	4 (0.0%)
No	125 (20.2%)			
Do not know	29 (4.7%)		9176 (68.8%)	
*Self-rated oral health*				
Very good	0 (0%)	22(3.4%)	124 (1.0%)	4 (0.0%)
Good	29 (4.7%)		1153 (8.6%)	
Moderate	149 (24.0%)		7100 (53.2%)	
Bad	282 (45.5%)		4255 (31.9%)	
Very bad	160 (25.8%)		709 (5.3%)	

*KNHANES* Korea National Health and Nutrition Examination Survey, *SD* standard deviation

### Prevalence of untreated caries and mean number of epidemiological indices of dental caries

The prevalence of untreated caries and the mean number of epidemiological indices of dental caries by age group in the inmate and KNHANES groups are summarized in Table 3.

**Table 3:** Prevalence of untreated caries and mean number of epidemiological indices of dental caries by age group

Age group (years)	Prevalence (rate) of untreated caries		DMFT		DT		MT		FT	
	Inmates n (%)	KNHANES n (%)	Inmates Mean (SD)	KNHANES Mean (SD)	Inmates Mean (SD)	KNHANES Mean (SD)	Inmates Mean (SD)	KNHANES Mean (SD)	Inmates Mean (SD)	KNHANES Mean (SD)
14–19	5 (41.7%)	196 (21.8%)	3.9 (4.5)	3.7 (4.1)	1.1 (1.8)	0.6 (1.4)	0 (0)	0.3 (1.7)	2.8 (3.7)	2.8 (3.5)
20–29	101 (70.1%)	451 (31.5%)	7.6 (4.9)	6.2 (4.5)	2.3 (3.0)	0.8 (1.5)	1.2 (2.0)	0.6 (1.7)	4.0 (3.8)	4.9 (4.1)
30–39	103 (74.1%)	664 (31.6%)	9.5 (5.0)	7.4 (4.6)	2.3 (2.6)	0.8 (1.6)	2.8 (2.4)	1.0 (2.4)	4.4 (3.9)	5.6 (4.2)
40–49	79 (58.1%)	621 (25.7%)	10.2 (6.0)	6.7 (4.9)	1.6 (2.5)	0.6 (1.3)	4.8 (4.0)	1.3 (2.7)	3.8 (4.2)	4.8 (4.1)
50–59	79 (60.8%)	591 (23.7%)	11.0 (6.1)	6.9 (5.0)	2.3 (3.7)	0.5 (1.3)	5.7 (4.1)	2.0 (3.4)	3.0 (3.6)	4.3 (3.9)
60–69	36 (54.5%)	508 (22.5%)	12.1 (6.6)	7.9 (5.8)	1.9 (3.5)	0.5 (1.3)	7.5 (5.4)	3.4 (5.1)	2.8 (3.2)	4.0 (3.8)
70–79	5 (38.5%)	416 (23.8%)	13.4 (5.1)	9.9 (6.9)	1.0 (1.6)	0.6 (1.5)	8.7 (5.2)	5.5 (6.9)	3.7 (3.6)	3.8 (4.0)
Total	408 (63.8%)	3447 (25.8%)	9.8 (5.9)	7.2 (5.4)	2.1 (3.0)	0.6 (1.4)	3.7 (3.8)	2.2 (4.3)	4.0 (4.1)	4.4 (4.0)

*DMFT* decayed, missing or filled teeth, *DT* decayed teeth, *MT* missing teeth, *FT* filled teeth, *SD* standard deviation, *KNHANES* Korea National Health and Nutrition Survey

### ***Prevalence of untreated caries and epidemiological indices of dental caries by self-reported questionnaire responses in the inmate group (Table ******4******)***

**Table 4:** Prevalence of untreated caries and epidemiological indices of dental caries by variables of interests

Inmates	Prevalence of untreated caries (Yes = 1, No = 0)		DMFT		DT		MT		FT	
	Beta	*p* Value	Beta	*p* Value	Beta	*p* Value	Beta	*p* Value	Beta	*p* Value
Sex (Men = 1, Women = 2)	− 0.548	0.147	0.046	0.654	− 0.393	0.094	− 0.003	0.983	0.319	0.101
Age (Years)	− 0.027	** < 0.001**	0.011	** < 0.001**	− 0.011	**0.010**	0.044	** < 0.001**	− 0.008	0.061
Time since last dental visit (Month)	0.009	**0.012**	0.001	0.111	0.004	** < 0.001**	0.000	0.726	− 0.004	** < 0.001**
Diabetes (Yes = 1, No = 0)	− 0.357	0.248	0.010	0.907	− 0.149	0.401	0.110	0.327	− 0.143	0.393
Cardiovascular disease (Yes = 1, No = 0)	0.078	0.832	0.031	0.752	0.290	0.163	0.134	0.316	− 0.350	0.084
Discomfort while chewing (Yes = 1, No = 0)	0.275	0.373	− 0.020	0.805	− 0.056	0.745	− 0.086	0.474	0.080	0.613
Bleeding gums (Yes = 1, No = 0)	− 0.519	0.071	− 0.162	**0.026**	− 0.323	**0.031**	− 0.085	0.423	− 0.056	0.693
Previous training on tooth brushing (Yes = 1, No = 0)	− 0.466	**0.026**	− 0.017	0.761	− 0.371	** < 0.001**	− 0.048	0.548	0.177	0.101
Number of times teeth are brushed in a day	0.163	0.164	0.007	0.816	0.109	0.082	− 0.039	0.369	− 0.015	0.805
Brushing of teeth before sleep (Always = 4, Usually = 3, Sometimes = 2, Rarely = 1)	− 0.096	0.411	0.005	0.870	− 0.018	0.777	0.025	0.570	0.052	0.394
Use of dental floss (Always = 4, Usually = 3, Sometimes = 2, Rarely = 1)	0.031	0.756	0.003	0.895	− 0.019	0.724	− 0.033	0.382	0.063	0.211
History of dental pain (Yes = 1, No = 0)	− 0.817	**0.008**	− 0.178	**0.025**	− 0.078	0.640	− 0.201	0.081	− 0.196	0.202
Self-rated oral health (Very good = 1, Good = 2, Moderate = 3, Bad = 4, Very bad = 5)	0.858	** < 0.001**	0.186	** < 0.001**	0.583	** < 0.001**	0.295	** < 0.001**	− 0.150	**0.031**
*KNHANES*										
Sex (Men = 1, Women = 2)	− 0.299	** < 0.001**	0.139	** < 0.001**	− 0.318	** < 0.001**	− 0.055	0.100	0.288	** < 0.001**
Age (Years)	− 0.011	** < 0.001**	0.009	** < 0.001**	− 0.009	** < 0.001**	0.043	** < 0.001**	− 0.004	** < 0.001**
History of dental pain (Yes = 1, No = 0)	0.181	** < 0.001**	− 0.010	0.547	0.082	0.066	0.036	0.329	0.024	0.235
Self-rated oral health (Very good = 1, Good = 2, Moderate = 3, Bad = 4, Very bad = 5)	0.447	** < 0.001**	0.128	** < 0.001**	0.461	** < 0.001**	0.263	** < 0.001**	− 0.004	0.766

Binary logistic regression analysis for the prevalence of untreated caries and negative binomial regression analysis for DMFT, DT, MT, and FT

Bold *p* values indicate significance (*p* < 0.05)

*DMFT* decayed, missing or filled teeth, *DT* decayed teeth, *MT* missing teeth, *FT* filled teeth, *KNHANES* Korea National Health and Nutrition Examination Survey

DMFT and MT values increased among inmates with increasing age. In contrast, the prevalence of untreated caries and DT values decreased with age (prevalence of untreated caries, beta =  − 0.027, *p* < 0.001; DMFT, beta = 0.011, *p* < 0.001; DT, beta =  − 0.011, *p* = 0.010; MT, beta = 0.044, *p* < 0.001). In addition, the longer the time since the last dental visit, the higher the number of DT and prevalence of untreated caries and lower the number of FT (prevalence of untreated caries, beta = 0.009, *p* = 0.012; DT, beta = 0.004, *p* < 0.001; FT, beta =  − 0.004, *p* < 0.001). General health, perceived oral health, and oral health habits affected each epidemiological index of dental caries.

The prevalence of untreated caries decreased in inmates who had previous training on tooth brushing experience (beta =  − 0.466, *p* = 0.026) and a history of dental pain reported (beta =  − 0.817, *p* = 0.008) and increased with a poorly self-rated oral health (beta = 0.858, *p* < 0.001).

The DMFT index decreased in inmates with a history of dental pain (beta =  − 0.178, *p* = 0.025) or bleeding gums (beta =  − 0.162, *p* = 0.026); however, it increased in those with self-rated poor oral health (beta = 0.186, *p* < 0.001). The number of DT increased in inmates with self-rated poor oral health (beta = 0.583, *p* < 0.001); however, it was not related to pain history. Inmates with little knowledge about brushing their teeth and light bleeding on their gums had greater DT values (previous training on tooth brushing, beta =  − 0.371, *p* < 0.001; bleeding gums, beta =  − 0.323, *p* = 0.031). In addition, inmates with poor self-rated oral health had higher MT values, and inmates with better self-rated oral health had higher FT values (MT, beta =  − 0.295, *p* < 0.001; FT, beta =  − 0.150, *p* = 0.031).

### ***Prevalence of untreated caries and epidemiological indices of dental caries by self-reported questionnaire response in the KNHANES group (Table ******4******)***

The KNHANES group had higher DMFT and MT values and lower FT and DT values and prevalence of untreated caries with increasing age (prevalence of untreated caries, beta =  − 0.011, *p* < 0.001; DMFT, beta = 0.009, *p* < 0.001; DT, beta =  − 0.009, *p* < 0.001; MT, beta = 0.043, *p* < 0.001; FT, beta =  − 0.004, *p* < 0.001). Women had lower DT values and prevalence of untreated caries than men, with higher FT values. Similarly, women had a higher DMFT index than men (prevalence of untreated caries, beta =  − 0.299, *p* < 0.001; DMFT, beta = 0.139, *p* < 0.001; DT, beta =  − 0.318, *p* < 0.001; FT, beta = 0.288, *p* < 0.001). In addition, the prevalence of untreated caries increased in the KNHANES group with a history of dental pain compared with the inmate group (beta = 0.181, *p* < 0.001). The self-rated oral health was significantly associated with these indices, with a poorer self-rated oral health associated with higher DMFT, DT, and MT values and prevalence of untreated caries (prevalence of untreated caries, beta = 0.447, *p* < 0.001; DMFT, beta = 0.128, *p* < 0.001; DT, beta = 0.461, *p* < 0.001; MT, beta = 0.263, *p* < 0.001). The number of FT was not significantly associated with this self-rated oral health.

### Comparison of prevalence of untreated caries and epidemiological indices of dental caries between groups

The prevalence of untreated caries and epidemiological indices of dental caries were investigated in the inmate and KNHANES groups. A generalized linear model was used. As shown in Table 5, the prevalence of untreated caries, DMFT, DT, and MT values differed significantly between the inmate and KNHANES groups (prevalence of untreated caries, DMFT, DT, and MT, *p* < 0.001). Other common variables of interest (sex, age, history of dental pain, and self-rated oral health) were included in this multivariable analysis. However, the results were almost identical to those of KNHANES group, where the number of samples was much more dominant.

**Table 5:** Prevalence of untreated caries and epidemiological indices of dental caries by common interest variables

	Prevalence of untreated caries (Yes = 1, No = 0)		DMFT		DT		MT		FT	
	Beta	*p* Value	Beta	*p* Value	Beta	*p* Value	Beta	*p* Value	Beta	*p* Value
Group (Inmates = 1, KNHANES = 0)	1.115	** < 0.001**	0.364	** < 0.001**	0.747	** < 0.001**	0.816	** < 0.001**	− 0.079	0.096
Sex (Men = 1, Women = 2)	− 0.300	** < 0.001**	0.138	** < 0.001**	− 0.313	** < 0.001**	− 0.054	0.096	0.289	** < 0.001**
Age (Years)	− 0.011	** < 0.001**	0.009	** < 0.001**	− 0.009	** < 0.001**	0.043	** < 0.001**	− 0.004	** < 0.001**
History of dental pain (Yes = 1, No = 0)	0.156	** < 0.001**	− 0.017	0.267	0.071	0.092	0.021	0.544	0.018	0.378
Self-rated oral health (Very good = 1, Good = 2, Moderate = 3, Bad = 4, Very bad = 5)	0.460	** < 0.001**	0.129	** < 0.001**	0.465	** < 0.001**	0.262	** < 0.001**	− 0.012	0.330

Binary logistic regression analysis for the prevalence of untreated caries and negative binomial regression analysis for DMFT, DT, MT, and FT

Bold *p* values indicate significance (*p* < 0.05)

*DMFT* decayed, missing or filled teeth, *DT* decayed teeth, *MT* missing teeth, *FT* filled teeth

### Interaction effects between groups and common interest variables on the prevalence of untreated caries and epidemiological indices of dental caries

We investigated the variables of interest (sex, age, history of dental pain, and self-rated oral health) by checking the interaction effect within the group. For individuals with a history of dental pain, the interaction term was significant for the prevalence of untreated caries and DMFT (prevalence of untreated caries, *p* = 0.019; DMFT, *p* = 0.013). Finally, for individuals with self-rated oral health, the interaction term was significant for FT (*p* = 0.011) (Table 6).

**Table 6:** Prevalence of untreated caries and epidemiological indices of dental caries with interaction effects by groups

	Prevalence of untreated caries (Yes = 1, No = 0)		DMFT		DT		MT		FT	
	Beta	*p* Value	Beta	*p* Value	Beta	*p* Value	Beta	*p* Value	Beta	*p* Value
Sex (Men = 1, Women = 2)	− 0.215	** < 0.001**	0.139	** < 0.001**	− 0.318	** < 0.001**	− 0.055	0.098	0.288	** < 0.001**
Age (Years)	− 0.008	** < 0.001**	0.009	** < 0.001**	− 0.009	** < 0.001**	0.043	** < 0.001**	− 0.004	** < 0.001**
History of dental pain (Yes = 1, No = 0)	0.127	** < 0.001**	− 0.010	0.541	0.082	0.059	0.038	0.344	0.024	0.237
Self-rated oral health (Very good = 1, Good = 2, Moderate = 3, Bad = 4, Very bad = 5)	0.321	** < 0.001**	0.128	** < 0.001**	0.464	** < 0.001**	0.263	** < 0.001**	− 0.004	0.766
Group (Inmates = 1, KNHANES = 0)	0.960	**0.018**	0.313	0.186	0.322	0.587	0.729	0.169	0.873	**0.006**
Group × Sex	0.053	0.808	− 0.088	0.472	0.202	0.514	0.032	0.908	0.033	0.842
Group × Age	− 0.000	0.907	0.003	0.156	0.004	0.524	0.005	0.388	− 0.006	0.067
Group × History of dental pain	− 0.333	**0.019**	− 0.218	**0.013**	− 0.216	0.322	− 0.329	0.090	− 0.194	0.100
Group × Self-rated oral health	− 0.057	0.449	0.041	0.350	0.052	0.642	0.023	0.813	− 0.153	**0.011**
Group marginal effect		** < 0.001**		** < 0.001**		** < 0.001**		** < 0.001**		** < 0.001**

Binary logistic regression analysis for the prevalence of untreated caries and negative binomial regression analysis for DMFT, DT, MT, and FT

Bold *p* values indicate significance (*p* < 0.05)

*DMFT* decayed, missing or filled teeth, *DT* decayed teeth, *MT* missing teeth, *FT* filled teeth

## Discussion

In South Korea, KNHANES data are publicly available, allowing for their use in many oral health-related studies [25–28]. Comparison of our findings with verified national data would provide a more objective assessment of the current oral health situation in correctional institutions.

A comparison of the prevalence of untreated caries and epidemiological indices of dental caries between the two groups revealed some interesting findings (Table 5). First, the inmate group had a higher prevalence of untreated caries, DMFT, DT, and MT (prevalence of untreated caries, beta = 1.115, *p* < 0.001; DMFT, beta = 0.364, *p* < 0.001; DT, beta = 0.747, *p* < 0.001; MT, beta = 0.816, *p* < 0.001). These values may be more useful in identifying healthcare disparities. Second, the FT value was not significantly different between the two groups. This finding was unexpected because even if the epidemiological indices of dental caries are high, the general population has better access to appropriate treatments, whereas inmates supposedly have fewer opportunities for dental care.

The results regarding sex revealed significant differences in both groups. In the inmate group, there was no significance of each indicator, but in the KNHANES group, significance was noted with the exception of MT. However, no interaction effects were observed within the group regarding sex, and we should take into account that the population accommodated had a greater percentage of men.

Furthermore, regarding the history of dental pain, significant differences were observed in both groups. As shown in the results, the prevalence of untreated caries decreased according to the history of dental pain in the inmate group but increased in the KNHANES group. The decrease in DMFT with a history of dental pain was significant only in the inmate group (Table 4), which was confirmed to represent an interaction effect (Table 6). This suggests that the aspects of pain experience by the incarcerated patients is different from that of the general population; using complaints of pain as a reference index for judging oral health warrants additional consideration.

Some studies showed differences in self-perception of pain and caries in the presence of fear [29] and reported that untreated caries were related to psychological factors, such as sense of coherence [30]. Considering this, when evaluating pain experience, it is necessary to additionally consider the various confounders of the incarceration environment. For example, this study did not adjust for information on time of incarceration. Moreover, history of dental pain was collected considering that different time points (3 months vs. 1 year) could be a confounding factor. The past 3 months for the inmate the group might be impacted by their time in prison and the pain may have passed by the time of the self-report questionnaire. In both cases, the prevalence/rates could be underestimated.

In the inmate group, self-rated oral health was the most significant factor affecting the prevalence of untreated caries and DMFT, DT, MT, and FT values (Table 4). Since it is important to identify patients with a history of untreated caries, DT, and MT, self-rated oral health may help identify patient groups requiring treatment. Unfortunately, in correctional institutions, the credibility of inmates is sometimes questioned [31]; nevertheless, our findings demonstrate that the self-rated oral health of inmates is sufficiently reliable and should be assessed during patient screening. In the KNHANES group the result showed significance, with the exception of FT (Table 4). The relationships between prevalence of untreated caries, DMFT, DT, and MT and self-rated oral health were significant in both groups, and no interaction effects with the group were observed for the prevalence of untreated caries, DMFT, DT, and MT (Table 6). The relationship between FT and self-rated oral health was significant only in the inmate group, which was confirmed again as an interaction effect within the group. Therefore, evaluating for the presence of dental caries should involve self-rated oral health in inmates. Similarly, FT had a significant correlation with the self-rated oral health in inmates and may, therefore, be useful as another evaluation index.

South Korean correctional institutions provide limited medical care due to insufficient resources [32]. Therefore, resources should be carefully allocated to inmates requiring care. Dental triage guidelines are being developed in other countries [31]; however, there are no such plans in South Korea at this time. Moreover, screening to evaluate oral health at the admission stage has not yet been instituted. It is difficult to fully understand the oral health status of inmates because screening and oral examination are not routinely conducted and because they do not visit the clinic often due to various reasons such as cost. If these indicators are included when establishing the screening stage, the evaluation itself can arouse interest in oral health. If the corresponding indicators are high, they could be used as auxiliary indicators to predict that oral health may be poor prior to oral examination, or they could be used as an evaluation index of the need for intervention for active prevention. This study indicates that self-reported oral health by inmates may be a reliable indicator of their oral health condition to help allocate resources more efficiently.

In addition to the common variables investigated between the two groups, the variables of interest investigated only for inmates (time since last dental visit, previous training on tooth brushing, and bleeding gums) were related to dental caries. There was no correlation with the level of hygiene care from the answers provided. Therefore, it is not easy to refer to the level of hygiene care that was self-evaluated.

This study had several limitations. First, only one dentist (I. Hwang) performed the inmate examinations, and radiographic aids were not used. Therefore, the findings were not verified, and prosthetics and restorations that looked esthetically appropriate may have been erroneously evaluated. But considering this dentist’s expertise, we felt it was safe to assume that he would have accurately identified most restorations. In addition, for patients with a longer duration of tooth loss and a higher number of teeth lost, the cause of the tooth loss could not be identified as caries, and many teeth were recorded as “unknown.” The records of third molars were based on the patient’s history; therefore, they may be unreliable.

Furthermore, our data were collected between 2019 and 2020; however, the KNHANES VII data were collected from 2016 to 2018. There is a slight discrepancy in the time periods of data collection; hence, there may be differences in the trends.

This study was conducted retrospectively on patients seeking in-house dental care, similar to previous studies from other countries [33–35]. The participants in the inmate group were patients and were not representative of the whole inmate population. Therefore, it was not possible to determine if the oral health of the overall inmate population was poorer than that of the general population. However, our findings may be considered meaningful considering the need for careful allocation of resources in correctional institutions compared with the general population.

Oral health screening of all inmates is not currently performed at correctional institutions and is limited to inmates seeking treatment. This indicates the scope of developing various indicators for more intensive dental care for inmates visiting the dental clinic. Moreover, among them, self-rated oral health demonstrated the potential of a new powerful indicator.

Determining the extent of dental care in the presence of limited resources is controversial from a legal, medical, and ethical standpoint. Unfortunately, there are no guidelines or legislation regarding the scope of dental care in correctional institutions in South Korea, and the medical management guidelines for inmates only specify procedures to be conducted at outside hospitals. Consequently, it is often unclear to correctional institution dentists if adequate dental care is being provided. Using oral epidemiological investigations to identify the oral health status of patients is of paramount importance during the development of criteria and guidelines.

## Conclusion

This is the first study to investigate the oral health of inmates at correctional institutions in South Korea. The oral health of the inmate group was significantly poorer than that of the general group. Since DMFT, DT, MT, and FT values and prevalence of untreated caries in the inmate group were significantly related to their self-rated oral health, it will likely be useful for the evaluations of these patients. In summary, this study suggests that more resources should be devoted to oral health care in correctional institutes in the future.

## Supplementary Information

**Additional file 1. Supplementary Text 1**. Dental Records Form Used during Dental Appointment.

## Data Availability

The data that support the findings of this study are available on request from the corresponding author, Hee-Kyung Park. The data are not publicly available due to the nature of this research and per the restrictions of our IRB. KNHANES oral examination raw data are available from https://knhanes.kdca.go.kr/knhanes/eng/index.do.

## Abbreviations

ATIA: Administration and Treatment of Correctional Institution Inmates Act
DMFS: Decayed, missing, and filled surfaces
DMFT: Decayed, missing, and filled teeth
DT: Decayed teeth
FT: Filled teeth
KDCA: Korea Disease Control and Prevention Agency
KNHANES: Korea National Health and Nutrition Examination Survey
MT: Missing teeth
