# Supplementary material for: Prevalence of dental caries and associated factors of detention center inmates in South Korea compared with Korea National Health and Nutrition Examination Survey (KNHANES) respondents: a retrospective study
Source: BMC Oral Health. 2022 Sep 5;22:383. doi: 10.1186/s12903-022-02405-w (PMC9446535; doi:10.1186/s12903-022-02405-w)
Supplement: Supplementary file 1 — Additional file 1. Supplementary Text 1. Dental Records Form Used during Dental Appointment. [file 12903_2022_2405_MOESM1_ESM.docx]

**Appendix 1**

**Public Health Dentist**

Under the Military Service Act of South Korea, all adult men must undergo physical examination and participate in national defense obligations. A person who holds a medical or dental license may serve as a public health doctor or dentist for at least 3 years after completing basic military training instead of military service. The number of dental public health dentists deployed to correctional institutions across the country were 52 in 2020, nine in 2015–2017, 17 in 2018, and 32 in 2019. The sixfold increase in the quota of dental public health dentists over the last 3 years is meaningful; however, questions remain on whether dental manpower is efficiently deployed and properly allocated in Korea. Unfortunately, none of the correctional institutions in the country employ dental hygienists. Therefore, only nurses can legally help dentists, although it is questionable whether the number of nurses with sufficient dental experience is adequate.

**Seoul Detention Center**

The Seoul Detention Center had more than 2,800 inmates aged 14–78 years between October 2019 and February 2020. In this institution, a dental public health dentist serves as the resident dentist. Due to the lack of manpower at the nearby Seoul Eastern Detention Center, there is also a shortage of available personnel and resources at the Seoul Detention Center. Dental sessions are conducted four times per week at this detention center and involve basic checkups, periodontal treatment, and extractions. There are also one or two dental sessions per month during which, inmates can pay for care from a visiting external dentist.

**Seoul Eastern Detention Center**

The Seoul Eastern Detention Center, which is the subject of another investigation, housed approximately 2,500 inmates aged 16–79 years between October 2019 and February 2020. This institution does not have a resident dentist; therefore, the dental public health dentist working at the Seoul Detention Center visits twice per week to provide dental care. No sessions with external dentists are conducted at this institution. Though treatment at external medical institutions is sometimes required, the limitations in security personnel and resources make this process difficult.

Supplementary Text 1. Dental Records Form Used during Dental Appointment

| Dental Records  [Questions]  How long has it been since you visited the dentist? ( ) Month(s)  Do you currently have diabetes? 1. Yes 2. No  Do you currently have cardiovascular disease? 1. Yes 2. No  Have you been uncomfortable chewing in the last 3 months? 1. Yes 2. No 3. Don’t Know  Have you felt pain in the last 3 months? 1. Yes 2. No 3. Don’t Know  Have your gums bled during the last 3 months? 1. Yes 2. No 3. Don’t Know  How do you think your mouth condition is when you evaluate yourself?  1. Very Good 2. Good 3. Moderate 4. Bad 5. Very Bad  Have you ever learned how to brush your teeth? 1. Yes 2. No  How many times do you brush your teeth per day? ( ) times  How many times have you brushed your teeth right before going to bed in the last week?  1. Always (7 times) 2. Usually (4-6 times) 3. Sometimes (1-3 times) 4. Rarely (0 times)  How often do you use dental floss?  1. Always (7 times) 2. Usually (4-6 times) 3. Sometimes (1-3 times) 4. Rarely (0 times)  [Oral Examination (Dental Chart)]  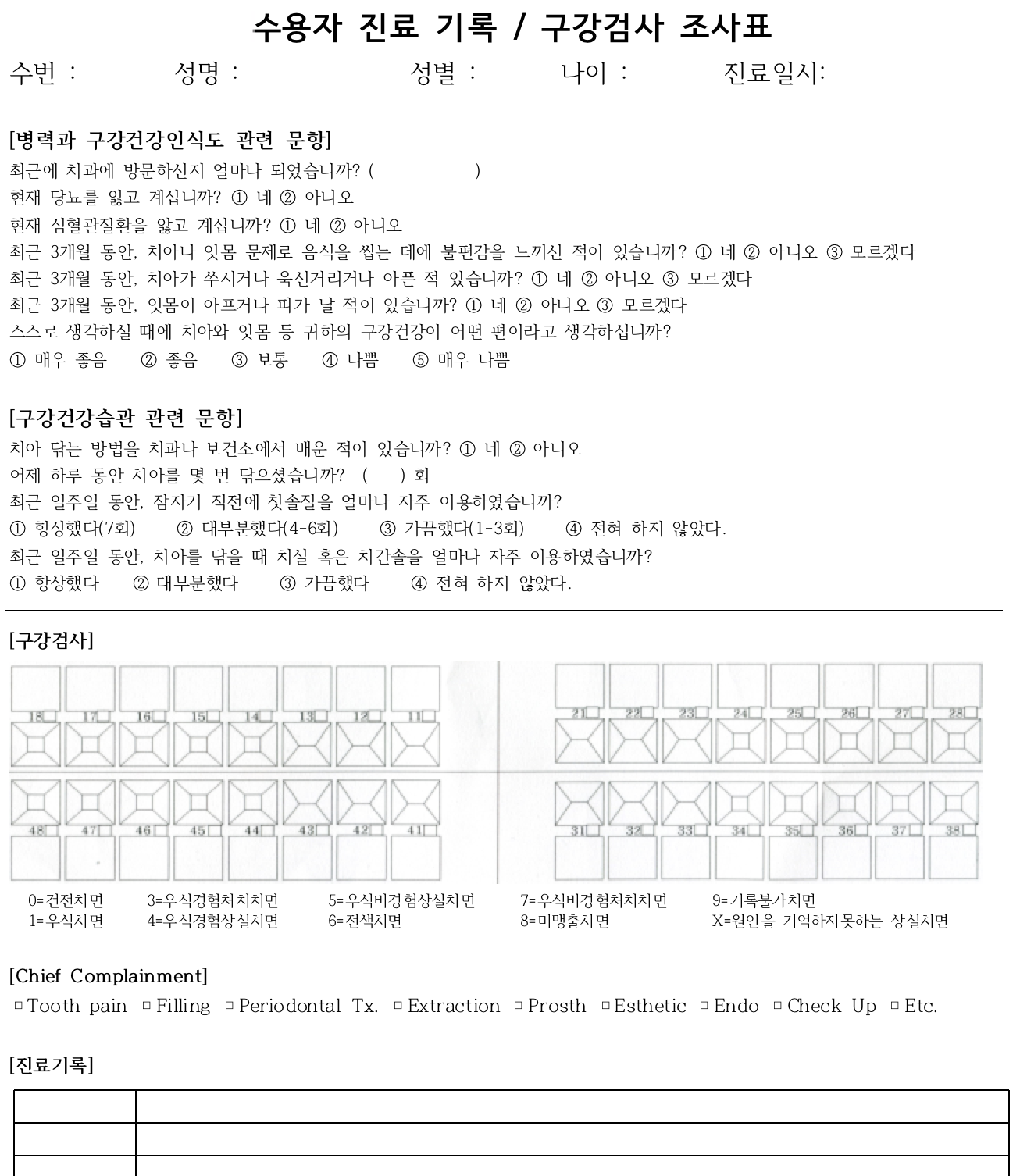  [Memo] |
| --- |
